# Case Commentary: A herculean effort for *Mycobacterium heraklionense*—localized azithromycin bead pharmacokinetics

**DOI:** 10.1128/aac.00305-25

**Published:** 2025-04-23

**Authors:** Shemual Tsai, Kevin Grimes

**Affiliations:** 1 Department of Pharmacy, Houston Methodist Hospital 23534 , Houston, Texas, USA; 2 Division of Infectious Diseases, Department of Internal Medicine, Houston Methodist Hospital 23534 , Houston, Texas, USA; Houston Methodist Hospital and Weill Cornell Medical College, Houston, Texas, USA

**Keywords:** mycobacterium, nontuberculous mycobacteria, azithromycin, pharmacokinetics, calcium sulfate beads, localized antibiotics

## Abstract

Non-tuberculous mycobacterial infections present significant treatment challenges due to resistance and limited data on optimal antibiotic regimens. A recently published case report provided an innovative approach to *Mycobacterium heraklionese* hand tenosynovitis with azithromycin-loaded calcium sulfate beads and valuable pharmacokinetic data with concentration measurements. This case demonstrates the ability of azithromycin-loaded calcium sulfate beads to achieve high local tissue concentrations with low systemic toxicity.

## COMMENTARY

Non-tuberculous mycobacteria (NTM) are widespread in the environment and can pose significant treatment challenges when pathogenic (1). Guideline-recommended antibiotic regimens for NTM typically require a prolonged course of multiple antibiotics due to intrinsic and acquired antibiotic resistance (2). NTMs can form biofilms and possess unique cell wall structures, such as with 3 → 3 peptide cross-linking, which limits the efficacy of β-lactam antibiotics. Additionally, resistance can emerge via spontaneous mutations that can lead to mechanisms such as upregulation of efflux pumps or β-lactamase production (3). Existing guideline recommendations for NTM are largely focused on certain NTM species such as *Mycobacterium abscessus* and *Mycobacterium avium* complex; hence, optimal antibiotic management for other NTM infections remains unclear, especially in musculoskeletal infections (2).

In this issue’s Challenging Clinical Case, Mikkelsen and colleagues present an innovative approach for managing an *M. heraklionense* hand tenosynovitis case. Six months after systemic antibiotic therapy was initiated, azithromycin-loaded calcium sulfate beads were implanted locally alongside a silicone rod insertion. Azithromycin concentrations in plasma and tissue (including tendon, muscle, tissue, and cancellous bone) were measured via microdialysis catheters. The use of azithromycin beads resulted in high local concentrations at the infection site with minimal systemic absorption. Although the efficacy of azithromycin-loaded beads for NTM infections remains uncertain, this case provides valuable insights into the pharmacokinetics of localized antibiotic therapy.

Azithromycin is known for extensive tissue distribution with its large volume of distribution and diprotic base molecular structure (4–6). Notably, azithromycin accumulates significantly in macrophages and neutrophils (5, 7). Intracellular azithromycin concentrations in white blood cells have been measured to exceed plasma levels by over 20-fold (8, 9). Given these properties, therapeutic drug monitoring of azithromycin plasma or tissue concentrations is not routinely performed.

In studies that have measured azithromycin concentrations, there is limited literature on pharmacokinetic targets for NTM infections (8, 10, 11). For *M. avium* complex (MAC) infections, peak azithromycin plasma concentration targets above 0.2 mcg/mL have been associated with favorable microbiological responses (12). The ratio of the 24 h area under the concentration-time curve to the minimum inhibitory concentration (AUC0-24/MIC) has also been proposed as a pharmacokinetic target for MAC, with values of 3.4 and 7.5 suggested for optimal efficacy and resistance suppression, respectively (13). Although these targets may not directly translate to *M. heraklionense* as in the case, it offers a preliminary benchmark. Mikkelsen and colleagues describe how the peak plasma and tissue concentrations exceed the proposed target. Additionally, tissue compartments in direct contact with the beads achieved nearly 100-fold higher concentrations than those not exposed. Although this suggests the potential for enhanced local drug delivery, optimal tissue concentration targets for NTM remain undefined. AUC_0-24_/MIC was not reported in this case and may be an opportunity for future studies to consider.

The utilization of calcium sulfate beads as the local delivery vehicle for azithromycin by Mikkelsen and colleagues adds a novel approach. Calcium sulfate beads offer several advantages, including prolonged antibiotic elution and sustained local antibiotic concentrations (14, 15). Although their use has been explored with glycopeptides and aminoglycosides in other infections, data on azithromycin-loaded beads for NTM remain limited (16–18). Intracellular azithromycin accumulation is pH-dependent, with protonation theorized to trap azithromycin and prolong its elimination half-life (4). Calcium sulfate bead dissolution generates an acidic environment, which may theoretically influence azithromycin concentrations and/or efficacy (19, 20). In this case, local concentrations near the azithromycin-loaded beads remained markedly higher than in other tissues and plasma. As azithromycin intracellular concentration was not measured, it would benefit future studies to assess whether this interaction may potentially affect intracellular concentrations, such as within lysosomes or white blood cells, which may be significant given the unique pharmacokinetic characteristics of azithromycin.

It is also important to remember the possible drawbacks of localized antibiotics. Mikkelsen and colleagues describe how the antibiotic calcium sulfate beads required removal after 10 days of implantation due to continuous, excessive wound drainage. Wound drainage is a well-documented complication of calcium sulfate, resulting from the acidity of beads dissolution (21–25). This may also increase infection risk, although findings are mixed (24, 26). Additionally, although antibiotic-loaded beads may have theoretically helped reduce the bacterial burden in the early stages, delayed wound healing has been described (20). As systemic azithromycin therapy is generally well tolerated, with doses up to 4 g being administered, it is important for clinicians to weigh the necessity of avoiding systemic exposure through local delivery against the potential risks of bead-related complications (27).

Fortunately, this approach appeared to be successful for this patient while providing insightful pharmacokinetic results. Nevertheless, the role of azithromycin-loaded calcium sulfate beads as adjunctive therapy for NTM infections remains speculative. It is unclear if an elevated tissue concentration, as noted, correlates with increased azithromycin efficacy. Risks associated with azithromycin bead placement should be carefully considered until more data on clinical outcomes are available. Further studies are needed to refine optimal dosing, bead volume, and target plasma, tissue, and intracellular concentrations. As macrolides remain integral to some NTM regimens, continued reporting of clinical experience with novel approaches like this case will be essential in shaping the future of NTM management.
